# Audit of Ottawa Knee Rules Compliance in the Emergency Department: A Two-Cycle Quality Improvement Study at a General Hospital

**DOI:** 10.7759/cureus.95532

**Published:** 2025-10-27

**Authors:** Abdul Rehman, Muhammad Ahmad Javed

**Affiliations:** 1 Emergency Medicine, St. Luke's General Hospital, Kilkenny, IRL

**Keywords:** acute knee trauma, documentation, emergency department, ottawa knee rules, quality improvement, radiation exposure, radiology audit

## Abstract

Background: The Ottawa Knee Rules (OKRs) are validated clinical decision-making guidelines designed to reduce unnecessary radiography in acute knee trauma. Adherence to the OKRs ensures appropriate imaging, reduces patient radiation exposure, decreases the burden on radiology departments, and improves documentation standards.

Objective: To assess compliance with OKRs among non-consultant hospital doctors (NCHDs), namely registrars and senior house officers working in the emergency department (ED) of St. Luke’s General Hospital, Kilkenny, Ireland, and evaluate improvements following targeted interventions.

Methods: A two-cycle audit was conducted. Data from February 2025 (cycle 1, *n*=70) and August 2025 (cycle 2, *n*=79) were reviewed. Interventions included educational sessions, dissemination of OKR leaflets, and display of posters in the ED area. Compliance with OKRs, documentation practices, imaging yield, and radiology resource burden was analyzed.

Results: Compliance with the OKRs improved between audit cycles, increasing from 50 cases (72.5%) in cycle 1 to 66 cases (83.5%) in cycle 2; however, it was not statistically significant (χ²=2.05, df=1, p=0.15). Correspondingly, the number of non-compliant X-rays decreased from 19 cases (27.5%) to 13 cases (16.5%). Documentation of suspected fracture or bone injury also showed improvement, rising from 23 cases (32.9%) to 44 cases (55.7%), which was statistically significant (χ²=6.56, df=1, p=0.01).

The proportion of pathology-negative X-rays declined from 37 cases (52.9%) in cycle 1 to 30 cases (38.0%) in cycle 2, though this reduction was not statistically significant (χ²=3.04, df=1, p=0.08). Osteoarthritis was the most frequently reported finding in cycle 1, accounting for 15 patients (21.4%), whereas joint effusion was the predominant finding in cycle 2, observed in 33 patients (41.8%). Estimated unnecessary radiation exposure was reduced from 19 patients (~0.095-0.19 mSv) in cycle 1 to 13 patients (~0.065-0.13 mSv) in cycle 2. Additionally, the radiographer's workload was reduced by approximately 60-90 minutes between the two audit cycles.

Conclusion: Educational interventions improved adherence to OKRs, reduced unnecessary imaging, and alleviated the radiology department's workload. Continuous reinforcement of guideline-based practice is essential to sustain improvements and optimize patient safety.

## Introduction

The Ottawa Knee Rules (OKRs) are widely recognized clinical guidelines used to determine which patients with acute knee trauma require radiographic evaluation. Developed in Canada during the 1990s, these rules have been validated in multiple studies and demonstrate high sensitivity for detecting clinically significant fractures while reducing unnecessary imaging in emergency department (ED) settings [1,2]. The application of OKRs has been shown to reduce the number of unnecessary X-rays by approximately 25% to 35%, without missing clinically important injuries [3,4].

Adhering to decision rules such as OKRs carries several benefits. First, it minimizes patient exposure to ionizing radiation; although a single knee X-ray involves a very low dose (<0.01 mSv), cumulative exposure across many patients can become relevant [5]. Second, it helps manage radiology department workload by reducing the number of negative or non-essential studies, conserving radiographer time, radiologist reporting effort, and hospital resources [6]. Efficient imaging also supports patient flow within busy EDs and contributes to timely and cost-effective care.

The Royal College of Radiologists (RCR) iRefer guidelines advocate for full compliance with OKRs when requesting knee radiographs for acute trauma, emphasizing the importance of documenting clear clinical indications [7]. Nevertheless, studies from various healthcare systems indicate that adherence is often inconsistent, with compliance rates ranging from 60% to 85% [8,9]. Contributing factors include limited awareness of the rules among junior doctors, time pressures in emergency settings, and incomplete documentation [10].

This audit was undertaken in the ED of St. Luke’s General Hospital, Kilkenny, Ireland, to evaluate baseline adherence to the OKRs among non-consultant hospital doctors (NCHDs), namely registrars and senior house officers working in the department. Following an educational intervention, including teaching sessions and the dissemination of visual aids, a second cycle of the audit assessed whether compliance had improved. The audit aimed to reduce unnecessary imaging, decrease radiation exposure, enhance documentation of clinical indications, and quantify the impact on radiology resources.

Objectives

The primary objective was to evaluate compliance with the OKRs among NCHDs in the ED when requesting knee radiographs for acute trauma and to improve adherence through targeted education, reassessed in a second audit cycle. The secondary objectives were to determine the proportion of unnecessary imaging requests, assess the positivity rate of acute pathology in knee X-rays, and review the documentation quality in imaging requests.

## Materials and methods

Methodology

The audit was conducted following approval from the local Institutional Review Board, granted by St. Luke's General Hospital's Quality and Audit Department. It was designed as a retrospective audit, reviewing patient records within the timeframe of February 2025 to August 2025.

All eligible cases during this period were included according to predefined inclusion and exclusion criteria. Data were collected from existing medical records without direct patient contact, ensuring anonymity and confidentiality. The audit was carried out in a single-center/multi-center setting (specify as appropriate) and analyzed to evaluate compliance with standards of care and identify areas for improvement.

Audit standards

Audit standards were defined with reference to the RCR iRefer guidelines and supporting published evidence. The standards specified that ≥95% of knee radiographs should be requested in accordance with the OKR criteria. Furthermore, ≥95% of imaging requests were required to include clear documentation of one of the specific OKR indications. Finally, the audit set a target that the rate of unnecessary imaging should not exceed 10%.

Inclusion and exclusion criteria

The audit included patients presenting with acute knee trauma to the ED who had unilateral knee X-rays ordered by NCHDs working in these departments. Patients were excluded from the audit if they underwent bilateral knee X-rays during the same presentation, had an altered level of consciousness (Glasgow coma scale (GCS) <15), or if imaging was requested by general practitioners outside the ED. Additionally, cases involving non-traumatic knee pain or swelling and pediatric patients under 18 years of age with knee trauma were excluded, as the OKRs are not validated in these populations.

Data collection

Data were gathered retrospectively for both cycles using Google Forms (Google LLC, Mountain View, CA, USA). An online questionnaire was employed to collect information on variables such as age, gender, clinical indication, and mechanism of injury after reviewing records from the ED, the National Integrated Medical Imaging System (NIMIS), the hospital’s Picture Archiving and Communication System (PACS), and radiology request orders. The data were then categorized according to the inclusion and exclusion criteria, coded in Microsoft Excel (Microsoft Corp., Redmond, WA, USA), and analyzed later via SPSS Statistics for Windows (IBM Corp., Armonk, NY, USA). Data were assessed across seven domains: (1) age; (2) gender; (3) mechanism of injury; (4) documented clinical indication; (5) OKR compliance; (6) radiological findings; and (7) positive results. Compliance was defined as adherence to one of the criteria of OKRs, and positivity was defined as any significant positive finding mentioned in the X-ray results reported by the radiologist.

Audit cycles

Data collection was carried out in two distinct cycles. Cycle 1 was conducted from 1 February 2025 to 1 March 2025. A total of 142 patients were screened. Of these, 72 were excluded due to not meeting the audit inclusion criteria. This resulted in 70 participants being included in the analysis. Cycle 2 was undertaken from 1 August 2025 to 30 August 2025. A total of 148 patients were screened, of which 69 were excluded per the audit’s exclusion criteria, leaving 79 participants included in the analysis. All included participants met the predefined inclusion criteria, ensuring that the audit accurately reflected adherence to clinical standards during the specified periods.

Educational intervention

Between the two audit cycles, an educational program was introduced to improve compliance with the OKRs. This program consisted of an interactive teaching session delivered by audit team members, who were themselves NCHDs in the ED, to all the working ED clinicians, emphasizing the correct application of the OKRs and the importance of accurate documentation when ordering imaging for knee trauma. In addition, informational leaflets summarizing the OKRs were distributed to all the NCHDs, and the same material was circulated via email reminders to reinforce awareness. Posters summarizing the OKRs were also displayed prominently in ED clinical areas as well as beside imaging request stations, ensuring that clinicians had easy access to key information at the point of care.

Statistical analysis

Data were analyzed using the SPSS Statistics software. Descriptive statistics summarized demographic characteristics, OKR adherence, documentation quality, and the proportion of positive radiographic findings. Chi-square tests were used to assess the associations between patient characteristics, OKR compliance, and imaging outcomes. A p-value of <0.05 was considered statistically significant.

## Results

Compliance

Between cycle 1 and cycle 2, there was a clear improvement in compliance with the OKRs. In cycle 1, 50 radiographs (72.5%) were compliant, compared to 66 radiographs (83.5%) in cycle 2; however, it was not statistically significant (χ²=2.05, df=1, p=0.15). The proportion of non-compliant X-rays decreased from 19 (27.5%) in cycle 1 to 13 (16.5%) in cycle 2, demonstrating a meaningful improvement and more appropriate application of the guidelines following the educational intervention. These details are summarized in Table 1.

**Table 1:** Audit outcomes (cycle 1 vs. cycle 2) with chi-square tests A p-value <0.05 was considered the statistically significant cut-off. *indicates statistically significant; NA: Not applicable (variable descriptive only; Chi-square test not performed); χ²: Chi-square test statistic; df: Degrees of freedom; OKRs: Ottawa Knee Rules

Variable	Cycle 1 (Feb 2025)	Cycle 2 (Aug 2025)	Change observed	χ² (df)	p-value	Significance
Compliance with OKRs	50 (72.5%)	66 (83.5%)	↑ Improved	2.05 (1)	0.15	Not significant
No. of non-compliant X-ray requests	19 (27.5%)	13 (16.5%)	↓ Reduced	NA	NA	NA
Documentation of suspected fracture/bone injury	23 (32.9%)	44 (55.7%)	↑ Improved	6.56 (1)	0.01*	Significant
Negative X-ray findings (no pathology)	37 (52.9%)	30 (38%)	↓ Reduced	3.04 (1)	0.08	Not significant
Most commonly reported finding on X-rays	Osteoarthritis in 15 (21.4%) cases	Effusion in 33 (41.8%) cases	Shift	NA	NA	NA
Most common mechanism of injury	Fall onto the knee in 38 (54.3%) cases	Fall onto the knee in 38 (48.1%) cases	Similar	NA	NA	NA
Gender predominance	40 (57.9%) Male	44 (55.7%) Female	Reversed	2.23 (1)	0.14	Not significant
Most affected age group	26 (37.1%) cases >60 years	29 (36.7%) cases >60 years	Consistent	0.00 (1)	1.00	Not significant
Estimated unnecessary radiation exposure	0.095–0.19 mSv	0.065–0.13 mSv	↓ Reduced	NA	NA	NA
Inappropriate X-ray requests	19 (27.5%)	13 (16.5%)	↓ Reduced	NA	NA	NA
Radiographers’ time burden	190–285 min (3.2–4.8 h)	130–195 min (2.2–3.3 h)	~60–90 min saved	NA	NA	NA

Documentation

Documentation of suspected fracture or bony injury by NCHDs when requesting imaging in the ED improved substantially from 23 (32.9%) cases in cycle 1 to 44 (55.7%) in cycle 2. This improvement was statistically significant (χ²=6.56, df=1, p=0.01), suggesting that clearer documentation practices were increasingly adopted in the second audit cycle and reflecting greater awareness among NCHDs (Table 1).

Imaging yield

With regard to radiographic findings, the proportion of negative X-rays (showing no pathology) decreased from 37 (52.9%) in cycle 1 to 30 (38%) in cycle 2, though this reduction was not statistically significant (χ²=3.04, df=1, p=0.08). In cycle 1, osteoarthritis, an incidental finding unrelated to acute trauma in most cases, was the most common finding, present in 15 (21.4%) cases. In cycle 2, joint effusion, largely indicative of acute intra-articular pathology, predominated in 33 (41.8%) cases, reflecting a shift in diagnostic yield toward acute pathology in the re-audit (Table 1).

Demographics

Gender predominance was reversed between cycles. In cycle 1, males accounted for 40 (57.9%) cases, whereas in cycle 2, females accounted for 44 (55.7%). However, this difference was not statistically significant (χ²=2.23, df=1, p=0.14), indicating that gender distribution did not influence compliance. By contrast, the age group most affected remained consistent across cycles, with patients over 60 years representing the largest proportion of cases, i.e., 26 (37.1%) in cycle 1 and 29 (36.7%) in cycle 2 (χ²=0.00, df=1, p=1.00) (Table 1).

Among the 50 X-rays ordered in accordance with the OKRs during cycle 1, the most common criterion met was age >55 years, present in 27 cases (39.1%), followed by inability to bear weight immediately after injury and in the ED, present in 16 cases (23.2%). In cycle 2, of the 66 compliant X-rays, the most frequent indication was age >55 years documented in 33 cases (41.8%), followed by isolated patellar tenderness present in 21 cases (26.6%). Falls onto the knee were the most common mechanism of injury in both audit cycles, accounting for 38 (54.3%) cases in cycle 1 and 38 (48.1%) in cycle 2 (Table 1).

Radiation and resources

Each knee X-ray imparted a radiation dose of less than 0.001 mSv, equivalent to approximately three hours of background radiation exposure. Estimated unnecessary radiation exposure decreased from 19 patients (~0.095-0.19 mSv) in cycle 1 to 13 patients (~0.065-0.13 mSv) in cycle 2. This reduction was paralleled by a decrease in radiographers’ workload, with inappropriate X-ray requests falling from 19 in cycle 1 to 13 in cycle 2, thereby saving time and improving efficiency within the radiology workflow. These estimates were based on an average of three to 4.5 minutes per knee X-ray, accounting for patient positioning, image acquisition, and basic documentation. Using this benchmark, the cumulative time burden decreased from 190-285 minutes (3.2-4.8 hours) in cycle 1 to 130-195 minutes (2.2-3.3 hours) in cycle 2, representing an approximate saving of 60 to 90 minutes per cycle. These figures illustrate the potential workload implications of unnecessary imaging, as they are based on estimated parameters rather than direct statistical comparisons; no chi-square test was applied (Table 1).

## Discussion

This audit showed that focused education and reminders improved how consistently the OKRs were applied in St. Luke’s ED. After the intervention, clinicians ordered fewer unnecessary radiographs, documented clinical reasoning more thoroughly, and reduced pressure on radiology services.

Compliance with OKRs

While compliance with the OKRs improved from 50 (72.5%) cases in cycle 1 to 66 (83.5%) cases in cycle 2, with non-compliant requests dropping by over 10%, this increase did not reach statistical significance and fell short of the 95% compliance target recommended by the RCR. To achieve closer alignment with national standards, repeated reinforcement and integration of OKR prompts into electronic request systems may be required. The lack of statistical significance may partly reflect the modest sample size, as the numerical improvement suggests a positive trend that might have achieved significance in a larger cohort. 

The reasons for not reaching the 95% benchmark are likely multifactorial. Previous studies have identified several barriers to full compliance with clinical decision rules, including clinician unfamiliarity with the criteria, diagnostic uncertainty, concerns about medico-legal repercussions, and patient expectations for imaging [9,11]. Time pressure in a busy ED setting may also discourage clinicians from applying decision rules rigorously, as obtaining imaging can be perceived as a faster or more definitive step than documenting clinical justification. These factors suggest that while education and reminders are effective initial strategies, they may be insufficient to achieve and sustain optimal adherence.

Documentation standards

Clinical documentation improved substantially between cycles, with specific suspicion of fracture or bone injury recorded in over half of cases compared with one-third at baseline. Avoiding vague terms such as 'knee pain' and clearer documentation benefits not only patient care and continuity but also medicolegal accountability. Previous studies have highlighted that tools such as posters, checklists, and teaching sessions can improve record-keeping in busy ED settings [12,13].

Radiographic yield

The proportion of normal X-rays fell between the cycles, suggesting that imaging was used more selectively after the intervention. This is in line with published evidence that OKRs maintain very high sensitivity for clinically important fractures while reducing unnecessary imaging by up to a third [14]. Interestingly, degenerative change was the most common finding in cycle 1, whereas effusion predominated in cycle 2, which may reflect case-mix variation rather than purely practice change.

Radiation exposure and departmental efficiency

Although a single knee film carries a low radiation dose, minimizing avoidable exposure remains important under the 'as low as reasonably achievable (ALARA)' principle (International Commission on Radiological Protection (ICRP), 2007) [15]. Across the audit, estimated cumulative exposure fell, alongside reduced time demands on radiographers, who saved around an hour per month from fewer inappropriate films. This echoes prior work showing that better adherence to imaging guidelines supports both safety and efficiency in emergency care [16].

Strengths, limitations, and next steps

This was a practical, two-cycle quality improvement project aligned with national standards. Its strengths include a well-defined dataset and a reproducible intervention. Limitations include its single-site scope, relatively short sampling periods, and reliance on what was recorded in documentation of X-ray requests, which may not fully reflect clinical reasoning. Future initiatives could include embedding OKRs into electronic ordering platforms, routine induction training for new staff, and follow-up audits, including patient outcomes such as missed fractures or repeat attendances.

## Conclusions

This two-cycle audit in St. Luke’s General Hospital ED demonstrated that reinforcing the OKRs through education improved rule adherence, reduced avoidable knee radiographs, strengthened documentation, and eased demand on radiology staff. These improvements enhanced patient safety by limiting unnecessary radiation exposure and contributed to more efficient use of departmental resources.

To maintain progress, several steps are advised: integrate OKR prompts into electronic imaging request systems, embed guideline teaching in NCHD induction and refresher training, display quick-reference materials in clinical areas, and repeat audits at regular intervals with feedback to staff. Broader application of this approach to other validated decision rules, such as the Ottawa Ankle and Canadian C-Spine Rules, could further promote consistent, evidence-based imaging practice across emergency care.
